# Bibliometric Study of Obstetrics Articles Published in the Journal of the American Medical Association, 1997-2016

**DOI:** 10.7759/cureus.3448

**Published:** 2018-10-13

**Authors:** Kana Yamamoto, Akihiko Ozaki, Shuhei Nomura, Yuki Senoo, Izumi Yoshida, Yuto Maeda, Mutsuko Ohnishi, Tetsuya Tanimoto, Masahiro Kami

**Affiliations:** 1 Internal Medicine, Navitas Clinic, Tokyo, JPN; 2 Surgery, Teikyo University Graduate School of Public Health, Tokyo, JPN; 3 Epidemiology and Biostatistics, Imperial College School of Public Health, London, GBR; 4 Internal Medicine, Medical Governance Research Institute, Tokyo, JPN; 5 Medical Education and Simulation, Semmelweis University, Budapest, HUN; 6 Obstetrics and Gynecology, Kobe City Medical Center General Hospital, Hyogo, JPN

**Keywords:** advanced age, marriage, complication, infertility, bibliometric, human immunodeficiency virus

## Abstract

Introduction

A recent increase in cases of advanced maternal age in the US has been partly associated with a higher incidence of pregnancy-related complications and infertility. However, little is known on how such social changes may have influenced obstetrics articles published in high-impact medical journals subscribed by diverse physicians. The objective of this study is to elucidate the presence and trend of obstetrics investigations in high-profile medical journals.

Material and methods

This bibliometric study retrospectively analyzed original articles published in the Journal of the American Medical Association (JAMA) from 1997 to 2016. Two reviewers extracted obstetrics articles from PubMed, assessed whether to include specific articles, and categorized them by subtopic. Main outcomes measure was the annual number of original investigations in obstetrics divided by that of original investigations from all fields during the study period, expressed as a trend.

Results

A total of 3486 original investigations were published during the study period. Regarding obstetrics, 1989 articles were originally extracted from PubMed; after a two-step review process, 199 (10.0%) obstetrics-related original investigations remained. Among them, 134 (67.4%) were classified as pregnancy-related abnormalities or complications (non-infection). The proportion of obstetrics articles decreased during the first 10 years but increased in the last 10 years. The highest figures in the first 10 and last 10 years were 8.5% in 1999 and 9.4% in 2014, respectively, whereas the lowest was 1.4% in 2008. The proportion articles on pregnancy-associated complications or abnormalities (non-infection) steadily increased during the study period, that of articles on infertility increased, and that of articles on human immunodeficiency virus (HIV) infection steadily decreased.

Conclusions

The observed trend may suggest a changing interest in obstetrics investigations among general physicians in the last 20 years. What is particularly notable is a heightened presence of research on pregnancy-related complications and infertility, which may reflect an increasing frequency in advanced maternal age in the US.

## Introduction

Women have tended to pursue careers more proactively in recent years [1], and a marriage and pregnancy at an advanced age has become more prevalent in high-income countries [2]. Advanced maternal age may be associated with infertility as well as other early (e.g., spontaneous abortion, chromosomal and gene abnormalities, and ectopic pregnancy) and late pregnancy issues (e.g., gestational hypertension, gestational diabetes, and other placental problems) [3, 4].

Under such circumstances, even general physicians may be required to acquire more knowledge about the latest progress in obstetrics, trends that might be reflected in high-profile general medicine journals. Given that the Journal of the American Medical Association (JAMA) is one of the most influential international general medicine journals, it would be reasonable to consider that its content reflects the general interests of physicians. To elucidate the chronological changes of 1) the presence of obstetrics research in all medical fields and 2) specific subtopics covered in obstetrics, we performed a bibliometric study of the original articles published in JAMA over the past 20 years.

## Materials and methods

We retrospectively abstracted all articles categorized as original investigations published in JAMA between January 1, 1997 and December 31, 2016 by referring to the online and print versions of the journal. We defined obstetrics as a branch of medicine that focuses on childbirth and maternal health [5]. In regard to original investigations related to obstetrics, we first extracted all relevant articles in JAMA using PubMed. Our search strategy is described in Supplementary Material 1.

In the first review, two authors (KY and YS) independently assessed the titles and abstracts of the extracted articles and judged whether the respective article should be included in this study. In the second review, the two authors repeated an independent assessment of the eligible articles, including full texts, and a final list of original obstetrics investigations was determined. At the third review, the two authors independently categorized each obstetrics article into one of the following subtopics without redundancy: “abnormality or complications in pregnancy (HIV-infection/non-HIV infection/non-infection),” “infertility,” “epidemiology,” and “others.” These subcategories were selected based on the thorough discussion among the authors, which was performed before the inception of the study. Disagreements in the categorization of specific article after each review round were resolved through discussion between the two authors, and one chief topic was chosen as a subcategory. The study period was divided into the following four quarters: first quarter, 1997–2001; second quarter, 2002–2006; third quarter, 2007–2011; and fourth quarter, 2012–2016.

The primary outcome measure was a ratio of the annual number of original obstetrics investigations divided by those of all fields in JAMA and its chronological change. The secondary outcome measure was a trend of subtopics covered in the obstetrics articles. The numbers of original articles in first quarter, second quarter, third quarter, and fourth quarter were 940, 984, 753, and 809, respectively. The numbers of original obstetrics investigations in first quarter, second quarter, third quarter, and fourth quarter were 66, 47, 31, and 55, respectively. All statistical analyses were performed using STATA 14.0.

## Results

**Figure 1:**
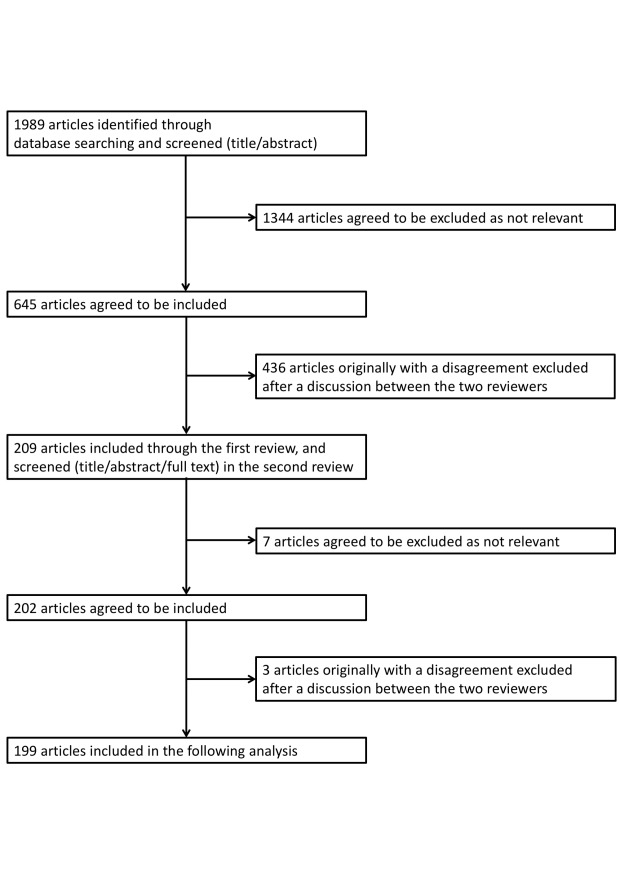
Original obstetrics investigations selection process.

A total of 1989 articles were initially extracted. Following the initial and second review processes, 209 and 199 articles remained as original obstetrics investigations, respectively. The inter-review kappa static was 0.30 and 0.34 at the first and second review, respectively. The total number of original investigations during the study period was 3486; of them, 5.4% detailed obstetrics. The process of article selection is described in Figure 1.

In the third review of 199 articles, 134, 14, 13, 12, 7, and 19 articles were classified as “abnormality or complications in pregnancy (non-infection),” “abnormality or complications in pregnancy (HIV infection),” “abnormality or complications in pregnancy (non-HIV infection),” “epidemiology,” “infertility,” and “others,” respectively. The inter-reviewer kappa static was 0.84, suggesting high reproducibility.

**Figure 2:**
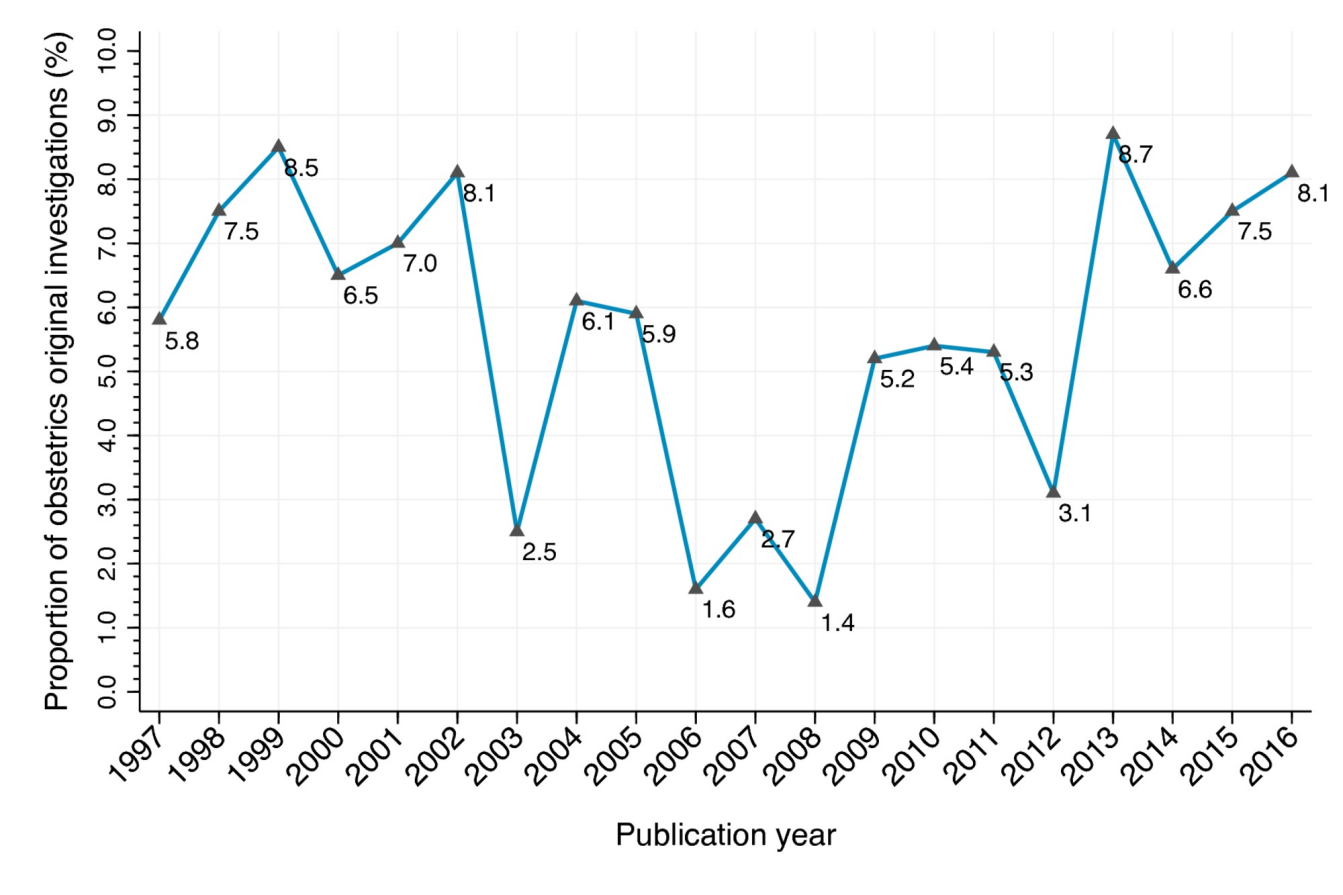
Ratio of annual original obstetrics investigations and all original investigations published in JAMA, 1997–2016.

As shown in Figure 2, the yearly trend curve of the proportion divided obstetrics original investigations by all original investigations published in JAMA, 1997-2016 showed a U-shaped transition; the figure gradually decreased during the first and second quarters (the highest in this period was 8.5% in 1999) and was lowest (1.4%) in 2008, while it gradually increased during the third and fourth quarters (the highest figure was 8.7% in 2013).

**Figure 3:**
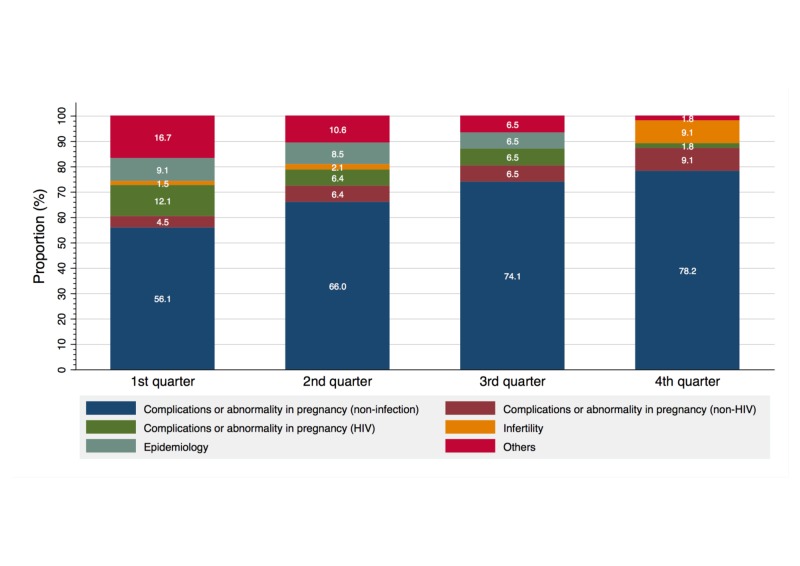
Trend of subtopics covered in obstetrics investigations, 1997–2016.

Figure 3 shows a trend of subtopics covered by the obstetrics articles. Throughout the study period, a proportion of the articles categorized as “abnormality or complications in pregnancy (non-infection)” was the highest among the subtopics. Further, this proportion steadily increased from 56.1% in the first quarter to 78.2% in the fourth quarter. Additionally, regarding “infertility,” while the proportion of articles covering this topic was 1.5%, 2.1%, and 0% in the first, second, and third quarter, respectively, it increased to 9.1% in the fourth quarter. In contrast, the proportion of articles on “abnormality or complications in pregnancy (HIV infection)” steadily decreased from 12.1% in the first quarter to 1.8% in the fourth quarter.

## Discussion

During the 20-year study period, obstetrics articles accounted for 5.4% of all articles published in JAMA. JAMA designates as many as 56 medical domains, including obstetrics, as its subject areas. Although we did not assess how much proportions other medical domains accounted for in the same journal, it may be reasonable to assume that obstetrics care is one of the most important subjects for both obstetricians and general physicians globally, but mainly in the US, where the journal’s editorial office is located. The proportion of obstetrics articles was relatively high in the first (1997–2001) and fourth (2012–2016) quarters of the study period but showed a decrease during the second (2002–2006) and third (2007–2012) quarters. This chronological change could be partly explained by the fact that the number of articles on HIV infection were steadily decreasing, whereas there was an increasing trend in those on infertility and pregnancy complications in women of advanced age. A possible reason for this phenomenon is that the standard HIV treatment for maternofetal infection is relatively established [6] and the number of practice-changing studies have gradually decreased, although this topic has continuously drawn attention from the funding bodies [7], whereas the importance of studies on the latter fields may have increased as a reflection of the social changes in a women’s status [8]. Further, because of the implementation of the Affordable Care Act and an increase in the number of retail clinics, women’s access to health care may be enhanced, a circumstance demanding up-to-date obstetrics knowledge even among general physicians [9].

Another interesting component is that the time frame of articles on infertility was increased only in the fourth quarter. Until recently, information on cutting-edge reproductive technologies such as in vitro fertilization (IVF) and intracytoplasmic sperm injection may have been shared only among obstetricians [10]. However, the use of infertility treatments has recently increased, and the frequency of births by IVF has drastically increased from 0.01 per 1,000 births in 1982 to 21.36 per 1,000 births in 2007 [11]. Given the continuous progression of social participation among women, such trends may have lasted until now. As a result, knowledge about infertility treatment would be widely required, even for general physicians [12].

There are several limitations in this study. First, we reviewed only articles published in JAMA, and the generalizability of the study is restricted. Indeed, given that the editor-in-chief of JAMA changed twice during the study period (2000 and 2011), it is possible that editor preference may have some influence on the current findings, which we did not consider these effects in our analysis. Second, a reproducibility of the two-step review processes to determine original investigations on obstetrics was fair, as suggested by the kappa coefficient of 0.30 and 0.34. In our study, while one reviewer (KY) was a physician, the other (YS) was a medical school student. Thus, a difference of their knowledge and experience in obstetrics and other medical fields may have led to the discordant judgement about with or without including specific articles into this study.

These situations call for a novel approach to assess articles for a bibliometric study. Recently, there is an increasing application of artificial intelligence into medical researches. Thus, a use of these cutting-edge techniques could enable a more consistent and rapid judgement for a large number of articles and journals, enhancing the generalizability of the findings in this type of studies. To date, several questions remain to be answered such as whether there were any differences within general medicine journals and between general medical journals and those specialized in gynaecology. We believe that a use of artificial intelligence could be one possible solution.

## Conclusions

In this retrospective bibliometric study of original articles published in JAMA, obstetric articles occupied a sizable proportion of all original articles. An increase in articles related to pregnancy complications and infertility and a decrease in those detailing HIV infection have mainly contributed to this chronological trend. These findings may reflect recent social changes including progress of late marriage, increases in elderly childbirth, and technological advances in the gynecology field.

## Appendices

Supplementary online material

Supplementary Material 1: Search Strategy for Article Selection on January 31, 2017

Search terms:

Reproductive Physiological Phenomena[MeSH Terms] OR "Congenital, Hereditary, and

Neonatal Diseases and Abnormalities"[MeSH Terms] OR "infant, newborn"[MeSH

Terms] OR "Pregnancy complications"[MeSH Terms] OR "Reproductive

techniques"[MeSH Terms] OR "Diagnostic Techniques, Obstetrical and

Gynecological"[MeSH Terms] OR "Delivery, obstetric"[MeSH Terms] OR

Infertility[MeSH Terms] OR "Abortion, induced"[MeSH Terms] OR "Obstetrics"[MeSH

Terms] OR "Obstetrics"[tiab] OR "Obstetric"[tiab] OR "Obstetrician"[tiab] OR

Obstetricians[tiab] OR "Obstetrical"[tiab] OR "Midwifery"[MeSH Terms] OR

midwifery[tiab] OR "midwives"[tiab] OR "midwife"[tiab] OR "Anesthesia,

Obstetrical"[MeSH Terms] OR "Analgesia, Obstetrical"[MeSH Terms] OR "Obstetric

Nursing"[MeSH Terms] OR "Obstetrical Forceps"[MeSH Terms] AND "JAMA"[journal]

AND (1997[PDAT] OR 1998[PDAT] OR 1999[PDAT] OR 2000[PDAT] OR 2001[PDAT] OR

2002[PDAT] OR 2003[PDAT] OR 2004[PDAT] OR 2005[PDAT] OR 2006[PDAT] OR

2007[PDAT] OR 2008[PDAT] OR 2009[PDAT] OR 2010[PDAT] OR 2011[PDAT] OR

2012[PDAT] OR 2013[PDAT] OR 2014[PDAT] OR 2015[PDAT] OR 2016[PDAT])
